# Complete Genome Sequence of Francisella guangzhouensis Strain 08HL01032^T^, Isolated from Air-Conditioning Systems in China

**DOI:** 10.1128/genomeA.00024-15

**Published:** 2015-03-19

**Authors:** Daniel Svensson, Caroline Öhrman, Stina Bäckman, Edvin Karlsson, Elin Nilsson, Mona Byström, Adrian Lärkeryd, Kerstin Myrtennäs, Per Stenberg, Ping-hua Qu, Johan Trygg, Holger C. Scholz, Mats Forsman, Andreas Sjödin

**Affiliations:** a Division of CBRN Security and Defence, FOI–Swedish Defence Research Agency, Umeå, Sweden; b Department of Chemistry, Computational Life Science Cluster (CLiC), Umeå University, Umeå, Sweden; c Department of Clinical Laboratory, Guangdong Provincial Hospital of Traditional Chinese Medicine, Guangzhou, China; d Department of Bacteriology and Toxinology, German Center for Infection Research (DZIF), Bundeswehr Institute of Microbiology, Munich, Germany

## Abstract

We present the complete genome sequence of Francisella guangzhouensis strain 08HL01032^T^, which consists of one chromosome (1,658,482 bp) and one plasmid (3,045 bp) with G+C contents of 32.0% and 28.7%, respectively.

## GENOME ANNOUNCEMENT

The known diversity of the genus Francisella has recently expanded substantially to include several new species. Genome analyses of representative Francisella isolates have shown that the genus can be divided into two main genetic clades (1). The first clade contains F. tularensis, F. novicida, F. hispaniensis, and Francisella-like endosymbionts (FLE), while the second clade contains F. noatunensis and F. philomiragia.

F. guangzhouensis type strain 08HL01032^T^ was isolated from air-conditioning systems in China in 2008 (2, 3). The live strain was obtained from the Public Health England Culture Collection (NCTC 13503) and assigned identifier FSC996 in the Francisella Strain Collection. The strain was grown on heart cysteine agar (HCA), and its DNA was extracted using standard techniques (4). Illumina HiSeq instruments generated a total of 17,862,240 paired-end reads (100 bp), with an average insert size of 540 bp, and 20,469,388 mate-pair reads (49 bp), with an average insert size of 4,927 bp. A Pacific Biosciences RSII system (10-kb library, 2-h movie length) generated a total of 90,148 PacBio reads, with an average read length of 3,575 kb, using two single-molecular real-time (SMRT) cells.

The initial draft of the genome was generated by assembling Illumina pair-end reads using the Edena version 3 assembler (5). Scaffolding was performed using Illumina mate-pair reads in SSPACE (6). The SMRT Analysis system version 2.2.0.p3 was used to assemble a second draft genome for PacBio reads. The two draft genomes were compared using progressiveMauve and MUMmer (7), and three regions of discrepancy were manually curated to generate the final assembly.

The final assembly consists of two scaffolds, one for the main chromosome and one for a circular plasmid. The main chromosome contains 1,658,482 bp with a G+C content of 32.0%, and the plasmid contains 3,045 bp with a G+C content of 28.7%. Annotation was carried out using the NCBI annotation service.

F. guangzhouensis strain 08HL01032^T^ contains 1,423 protein-coding sequences, 75 pseudogenes, 10 rRNAs, 38 tRNAs, and 1 noncoding RNA. The average nucleotide identity (ANI) was calculated by pairwise genome comparisons for publically available genomes within clade I and clade II (1) using JSpecies version 1.2.1 (8). The similarity between F. guangzhouensis and clade I genomes was 75.6% to 75.2% and to clade II genomes 80.0% to 74.8%, respectively. Commonly, a threshold of >95% to 96% identity is used to classify genomes as belonging to the same species (9). The plasmid was 89% identical over 1,408 bp to F. philomiragia plasmid pF242 (10). The phylogeny shows that F. guangzhouensis does not belong to any of the two previously known Francisella main clades or the recently published F. endociliophora clade (11). This isolate forms a new separate branching clade in the Francisella genus. The updated knowledge is essential for improving assays to be used in epidemiological studies of Francisella (12).

### Nucleotide sequence accession numbers.

This whole-genome shotgun project has been deposited at DDBJ/EMBL/GenBank under the accession numbers CP010427 (chromosome) and CP010428 (plasmid). The versions described in this paper are the first versions, CP010427.1 and CP010428.1.
